# Forest Fire Influence on *Tomicus piniperda*-Associated Fungal Communities and Phloem Nutrient Availability of Colonized *Pinus sylvestris*

**DOI:** 10.1007/s00248-022-02066-w

**Published:** 2022-07-13

**Authors:** Kerri Kluting, Ylva Strid, Diana Six, Anna Rosling

**Affiliations:** 1 Department of Ecology and Genetics, Evolutionary Biology, Uppsala University, 752 36 Uppsala, Sweden; 2 Department of Ecosystem and Conservation Sciences, University of Montana, Missoula, MT 59812 USA

**Keywords:** Bark beetles, Gallery, Larvae, Pine

## Abstract

Forest fire is known to positively affect bark beetle populations by providing fire-damaged trees with impaired defenses for infestation. *Tomicus piniperda*, the common pine shoot beetle, breeds and lays eggs under the bark of stressed pine trees and is considered a serious forest pest within its native range. Wood-colonizing fungi have been hypothesized to improve substrate quality and detoxify tree defensive chemistry to indirectly facilitate tree colonization by beetles. While some bark beetle species form symbiotic associations with fungi and actively vector their partners when colonizing new trees, *T. piniperda* does not have mycangia or body hairs for specific vectoring of fungi. To explore the *T. piniperda*-associated fungal community for signs of specific association, we used ITS metabarcoding to separately characterize fungal communities associated with surface and gut of male and female beetles. We also characterized the temporal changes in fungal community and nutrient status of pine phloem with and without beetle galleries. Sampling was performed 2 years after a natural forest fire and included both burnt and unburnt sites. In our study system, we find that forest fire significantly impacts the fungal community composition associated with *T. piniperda* and that fire may also indirectly change nutrient availability in phloem to beetle galleries. We conclude that *T. piniperda* can vector fungi to newly colonized trees but the absence of positive effects on substrate quality and minimal effects of sex indicate that vectoring of associated fungal communities is not a strategy associated with the *T. piniperda* life cycle.

**Supplementary Information:**

The online version contains supplementary material available at 10.1007/s00248-022-02066-w.

## Introduction

Most nutrients in plant tissues are present in relatively low concentrations compared to those found in the herbivores that feed upon them [1]. Therefore, herbivores must often consume considerable amounts of plant tissues to meet their nutritional needs. For insects, nitrogen (N) is often limiting, which can result in low growth rates and low breeding success [2, 3]. Phosphorous (P), which is required for producing a variety of essential compounds such as DNA, RNA, ATP, and proteins [4–7], can also be limiting for herbivorous insects. Insects contain approximately 6–10% N [8, 9] and around ten times higher P concentration than plants [9, 10], although these concentrations can vary greatly [11]. Tree tissues vary in how much nutrients (N and P) they contain relative to carbon (C), with leaves often containing the most nutrients and the bark and sapwood containing the least. Somewhere in between is the phloem [9, 12], the conductive tissue where photosynthates, amino acids [13], and polyphosphates are transported throughout the tree [14], and the main feeding substrate of bark beetles.

However, phloem is nutrient deficient enough that beetles feeding on it are challenged to meet their nutritional needs. Beetle larvae must either consume many times their weight in phloem or feed on fungi that translocate nutrients to the site of larval feeding and development [8, 9, 11, 15]. Some bark beetles form specific symbiotic relationships with fungi that transport limiting nutrients such as N and P from the sapwood to the phloem where beetle larvae feed [16, 17]. In some obligate mutualisms, beetles have specific structures called mycangia to transport their fungal partners when they fly to breed and colonize new trees, thus functioning as active vectors for these wood-colonizing fungi [17]. Bark beetle species that lack mycangia could potentially carry specific fungi or a variable suite of fungi [18–20] that can influence nutrient availability to beetles [11]. Wood-colonizing fungi that translocate nutrients from sapwood to phloem in order to support their own growth can provide by-product nutritional benefits to beetles even when the species are not tight associates [11]. Additionally, a need for maintaining a specific beetle–fungal association could be relaxed if a diverse fungal community can provision N and P to the beetles. For successful infestation and development in a tree, the beetle must also be able to overcome tree defenses. Various fungal species, such as those that are pathogenic, detoxify tree defensive chemistry and indirectly facilitate tree colonization by beetles [21, 22]. However, some bark beetle species are attracted to fire-damaged trees, which often have reduced defenses, including low resin flow [23, 24].

*Tomicus piniperda*, the common pine shoot beetle, breeds and lays eggs under the bark of stressed or recently killed pine trees [25, 26]. *Tomicus piniperda* may be the primary stem colonizers of trees and dominating species among stem-attacking bark beetles, in particularly in storm-felled pine trees [27]. The larvae feed in the phloem and then move into new shoots as juvenile adults. Mined shoots die and drop, causing reductions in tree growth [28, 29]. Fire-damaged pines are particularly susceptible to *T. piniperda* infestation and are usually attacked within 2 years post-fire [30]. Beetle populations can increase rapidly in fire-stressed trees or following storm fells. Reduced tree growth and rapid population growth has led to *T. piniperda* being considered a serious pest within its native range, which extends from the Palearctic region from Europe throughout Siberia to Japan [31–34]. While *T. piniperda* does not have mycangia, it is known to vector fungi to pine hosts [25, 35–37]. However, whether vectored fungal communities are consistent in space or time remains unknown.

Forest fire is known to positively affect beetle populations by providing fire-damaged trees with impaired defenses for infestation, but the indirect effects, such as changed nutrient availability, are not as well understood. Here, we aim to provide a more comprehensive overview of relationships between fire disturbance, beetle-vectored fungal communities, phloem nutrient quality, and changes in the gallery fungal community during larval development. We specifically address the specificity of *T. piniperda*-associated fungal communities and analyze their link to substrate quality in the phloem to identify possible specific beetle–fungal associations.

## Materials and Methods

### Sampling Locations

**Fig. 1:**
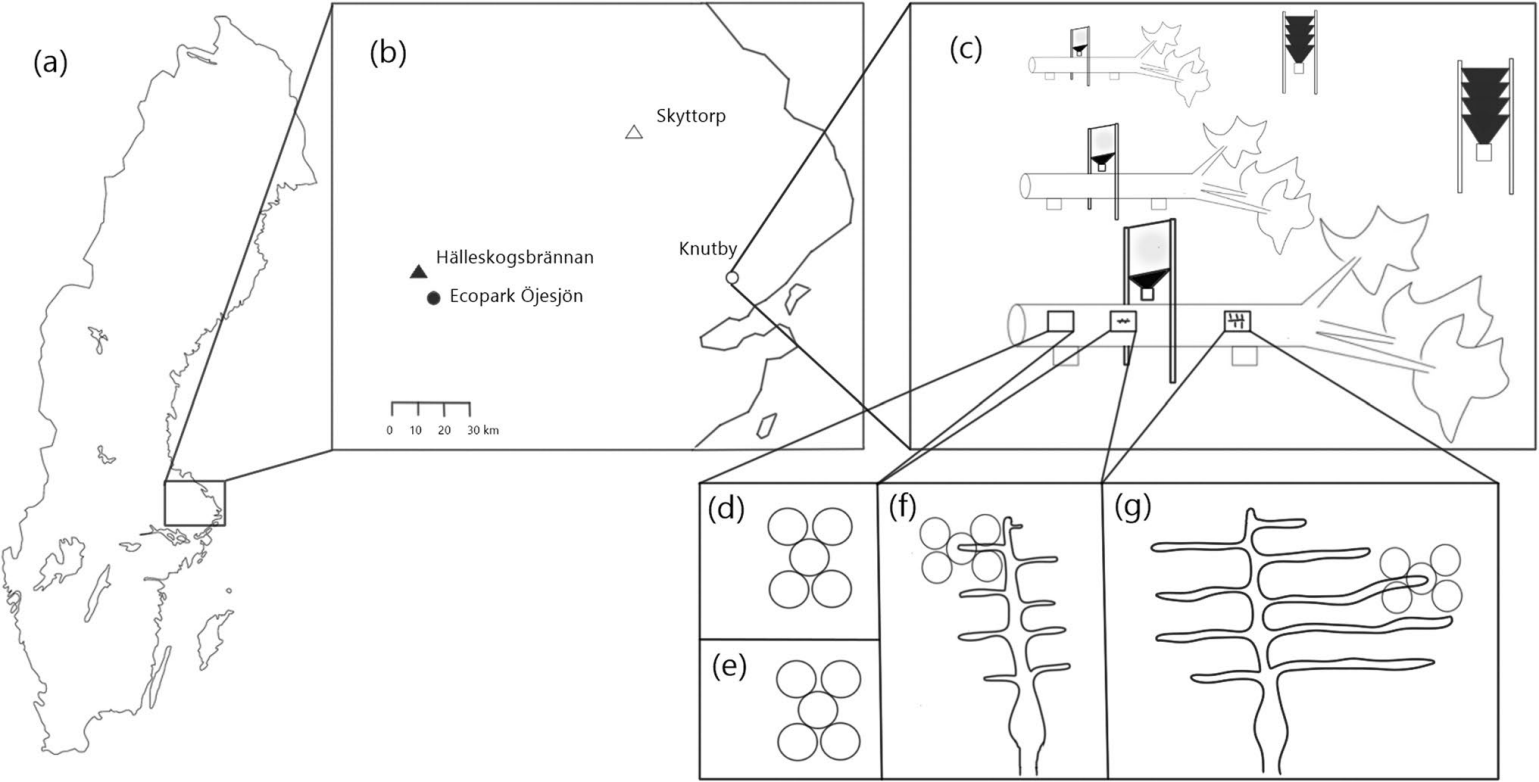
Overview of Sweden indicating the study area (a). Two of the sites (Hälleskogsbrännan and Ecopark Öjesjö) were affected by forest fire in 2014 (burnt sites represented by filled symbols), and two (Skyttorp and Knutby) were not (unburnt sites represent by open symbols) (b). At each site, three felled trees that were elevated from the ground were selected for this study (c). Window and funnel traps were mounted above or nearby the trees (c). At three time points, five phloem plugs were collected for carbon, nitrogen, and phosphorus concentration quantification and for DNA sampling (d–g). The first time point (d) was when the trees were newly cut, before the *Tomicus piniperda* flying season. Those pre-colonization samples and the control samples from the two post-colonization time points (e) (corresponding to the first and third instar stage of *T. piniperda* larvae in the phloem) had no connection to larval activity or galleries. Samples of larvae-colonized phloem were taken from the edges of beetle galleries first during the first instar larval stage (f) and again during the third larval instar stage (g)

For this study, four 20- to 30-year-old pine forest stands in Sweden were selected to explore the effect of forest fire on phloem nutrient quality and *T. piniperda-*associated fungal communities, Hälleskogsbrännan (HSB: 59°54′35.5″N, 16°08′42.2″E), Ecopark Öjesjön (OJ: 59°51′04.2″N, 16°14′57.8″E), Knutby (KB: 59°56′46.1″N, 18°18′00.4″E), and Skyttorp (SK: 60°06′32.4″N, 17°49′34.0″E) (Fig. 1). Two of the sites (HSB and OJ) were affected by a single forest fire event during summer 2014, and the other two were not (Fig. 1b). Following forest fire, substrate availability is high, and beetles do not fly far from these abundant food sources [38, 39]. Thus, we expect that the distance between HSB and OJ (approximately 9 km) is enough to assume that the bark beetle populations and associated fungal communities sampled from the two burnt sites are independent of each other.

### Sample Collection

The study was initiated in February 2016, at which time we expected *T. piniperda* to have established in the area and have completed one generation post-fire. Five trees at each site, spaced 30 m apart, were felled in mid-February, before the first *T. piniperda* flight. The trees were placed on plastic cylinders approximately 20 cm above the forest floor and left without additional treatment (Fig. 1c).

In early March before warming temperatures would initiate beetle flight, three free-hanging window traps were mounted above each of the stems, and two funnel traps were mounted by the felled trees at each site. Traps were baited with 70% ethanol and ( −)-α-pinene (Sigma-Aldrich, Saint Louis, MO, USA) to attract adult *T. piniperda* to the traps during flight (Fig. 1c). To avoid cross-contamination between beetles, an artificial pinecone made by carbon paper (Fig. S1) was placed in each collection cup so that the beetles could hide from cannibalism and other enemies while in the cup. To monitor beetles flight, traps were continuously checked from March 11–April 6, 2016. When beetles were observed in the cups, the artificial cone was transferred to a watertight plastic bag, and a new artificial cone was placed in the collection cup. Cones were handled separately and transported on ice to the lab where they were stored at 4 °C. Within 24 h, *T. piniperda* collected from each artificial cone were sorted by sex and dissected under a stereomicroscope. Samples were prepared by placing two individuals of the same sex into a 1.5-mL microcentrifuge tube. To collect spores and fungal propagules from their bodies and gut contents, the beetle samples were washed by vortexing for 30 s in 50 µL RNA*later*™ Stabilization Solution (Invitrogen, Carlsbad, CA, USA), and the resulting body wash solution was collected. Gut samples of both individuals were then collected by dissecting the beetles by splitting the back body of the insect using a forceps. The gut was put in RNA*later*™ Stabilization Solution (Invitrogen, Carlsbad, CA, USA). Samples were collected to represent all of the traps with beetles from each sampling occasion. Between two and four sampling occasions were needed to reach the target of 24 samples of each sex from each site. In the end, four different beetle sample types were thus collected: female body wash, male body wash, female gut, and male gut, all with 24 replicates per site for a total of 384 beetle samples. All samples were stored frozen until DNA extraction.

After felling, a phloem sample (pre-colonization time point) was taken from each of the stems using a leather punch (28 mm diameter) and a rubber hammer (Fig. 1d). Bark was removed prior to phloem sampling. Each sample was a composite of five phloem plugs placed in an air-tight plastic bag and transported on ice back to the laboratory where they were frozen at − 20 °C and later freeze-dried (Heto LyoLab 3000 Freeze-Dryer, Thermo Fisher Scientific, Waltham, MA, USA) for at least 24 h.

At each site, three of the five felled trees were chosen in April to be included in the study based on successful beetle colonization during beetle flight. This resulted in a total of 12 felled trees across the four sites. Following *T. piniperda* colonization, phloem samples were collected from each tree at two time points corresponding to two larval developmental stages. Two control samples were taken from areas ≥ 30 cm from the closest larval gallery (Fig. 1e) at both times, and eight phloem samples in galleries were taken in the first instar (mid-April; Fig. 1f), as well as in the third instar (mid-May; Fig. 1g). At both time points, a total of 10 phloem samples were collected from each felled tree (as described previously for pre-colonization samples). Each larval gallery represents a separate brood resulting from one infestation event. This resulted in a total of 252 phloem samples: ten samples (eight colonized and two uncolonized phloem samples) at two post-colonization time points from each of the 12 trees, as well as the sample taken pre-colonization from each tree. Just as with the pre-colonization phloem samples, each sample was a composite of five phloem plugs. All samples were transported on ice to the lab and frozen at − 20 °C within 12 h. Samples were later freeze-dried (Heto LyoLab 3000 Freeze-Dryer, Thermo Fisher Scientific, Waltham, MA, USA) for at least 24 h and stored for DNA extraction.

### DNA Isolation, Amplification, and Sequencing

Prior to DNA extraction, *T. piniperda* samples (body and gut) were homogenized in 2-mL screw cap tubes with three stainless steel hexagon screw M3 nuts (BAHAG AB, Mannheim, Germany) and approximately 40 borosilicate glass balls (1 mm diameter) (Sigma-Aldrich, Saint Louis, MO, USA) at a frequency of 30 Hz for 3 min in a bead beater (TissueLyser II, Qiagen, Hilden, Germany). Phloem samples were homogenized by grinding using a kitchen mixer at 16,000 rpm for 5–10 s (Bamix, Mettlen, Germany). DNA was extracted from the beetle samples using the NucleoMag Plant Kit (Macherey–Nagel, Düren, Germany). DNA extraction was conducted using the same kit for homogenized phloem samples, but with double the amount of lysis buffer due to the high absorption capacity of the phloem samples. The internal transcribed spacer 2 (ITS2) region of nuclear ribosomal DNA was amplified by PCR using the forward primer fITS9 (5′–GAACGCAGCRAAIIGYGA–3′) [40] and reverse primer ITS4 (5′–TCCTCCGCTTATTGATATGC–3′) [41], each with unique barcoded primers. Three parallel PCRs were conducted in 20 µL volumes containing 1.5 U DreamTaq DNA polymerase (Thermo Fisher Scientific, Waltham, MA, USA) with 10** × **DreamTaq™ buffer containing 20 mM MgCl_2_, 0.2 mM dNTP, 1 mM fITS9 primer and 0.3 mM ITS4 primer, and 10–20 ng genomic DNA template. MgCl_2_ concentration was optimized for each sample type to a final concentration of 22.75–29 mM. Reactions were carried out using an Applied Biosystems 2720 Thermal Cycler (Applied Biosystems, Foster City, CA, USA) with the following cycling protocol: an initial denaturation step at 95 °C for 10 min, 30–35 cycles of denaturation at 95 °C for 30 s, annealing at 58 °C for 30 s, and extension at 72 °C for 50 s, followed by a final extension step 72 °C for 3 min. Negative and positive controls were included in each PCR (DNA from an *Agaricus bisporus* fruitbody was used as the positive control). Products from the three parallel PCRs were pooled and amplification was confirmed via gel electrophoresis using a 1.5% Agarose Basic (PanReacc AppliChem ITW Reagents, Chicago, IL, USA) and GelRed Nucleic Acid Gel Stain (Biotium, Inc., Freemont, CA, USA). DNA from 423 of the 636 samples was successfully amplified. Amplified DNA was cleaned using the AMPure XP kit (Beckman Coulter, Brea, CA, USA) and then quantified using a Quant-iT PicoGreen dsDNA Assay Kit (Life Technologies, CA, USA). Equal amounts of PCR product from each sample, approximately 50 ng, were pooled into one library for body and gut samples and a separate library for phloem samples. The two libraries where vacuum centrifuged at 1300 rpm (Scan Speed 32, Labogene ApS, Lynge, Denmark) until the volume reached 60 µL and sequenced at SciLifeLab/NGI (Uppsala, Sweden) on a PacBio RS II system (Pacific Biosciences, Menlo Park, CA, USA) using two SMRT cells (one for beetle samples, one for phloem samples). Sequence data were delivered to us as error-corrected FASTQ files (containing circular consensus sequencing reads).

### Quantification of C, N, and P in Phloem Samples

From each homogenized phloem sample, approximately 12 mg was used to determine total C and N by combustion (Costech Elemental Combustion System 4010, Costech Analytical Technologies, Inc. Italy). Total P was quantified using an ICP AVIO200 (Perkin Elmer, Waltham, MA, USA) in nitric acid extracts following the national standard protocol SS 28,311:2017 (SIS, Stockholm, Sweden). Extraction and quantification of P were conducted in the certified laboratory at the Department of Soil and Environment, Swedish University of Agricultural Sciences, Uppsala, Sweden. Quantification of C and N was successful for 249 of the 252 phloem samples, and P concentration was successfully quantified for all samples. Phloem N, C, and P concentration (mg/kg) and C:N, C:P, and N:P ratio (molar) were calculated for each sample.

### Read Quality Filtering and OTU Generation

Sequence reads were quality filtered, demultiplexed, and clustered into Operational Taxonomic Units (OTUs) using the Sequence Clustering and Analysis of Tagged Amplicons (SCATA) pipeline (available at https://scata.mykopat.slu.se/ and first described in [40]. For this we used the following settings: clustering distance of 1.5% sequence similarity, minimum sequencing length threshold of 150 bp, and minimum allowed base quality of 2. After read clustering, all global singletons were removed. For simplicity, we will refer to cluster names “scata3997_number” as “OTU_number” throughout the text. Samples with invalid tagged primer combinations were removed from the resulting OTU/sample matrix, as well as any singletons created by their removal. The resulting OTU/sample matrix consisted of 15,854 reads (412 samples, 362 OTUs). Any OTUs with zero reads in the dataset were also removed from the FASTA file containing the representative sequence for each OTU cluster.

The ITS2 region was then extracted using ITSx (version 1.1.2) [42], and taxonomy prediction was accomplished using the SINTAX classifier (USEARCH v11.0.667; [43] with a bootstrap confidence cut-off of 0.8. The UNITE USEARCH/UTAX dataset (version 8.0, all eukaryotes, https://doi.org/10.15156/BIO/786346, UNITE [44] was used as the reference dataset and was modified following [45] so that it would be compatible with the SINTAX classifier [43]. Sequences for which the ITS2 region was not successfully extracted by ITSx or were not predicted to represent fungi were filtered out of the dataset for downstream analysis. In addition, sequence reads corresponding to *A. bisporus* (OTU_1) were removed from the dataset, since DNA from an *A. bisporus* fruitbody was used as the positive control for PCRs and this organism is unlikely to occur naturally in this environment. This resulted in a dataset consisting of 12,313 reads (401 samples, 289 OTUs). Samples with less than 10 reads were also excluded from downstream analyses, as well as any singleton OTUs in the dataset created by the removal of these samples, resulting in 11,962 reads (331 samples, 286 OTUs) used for analysis of fungal communities.

### Data Visualization and Multivariate Statistical Analysis

#### Fungal Communities

Non-metric multidimensional scaling (nMDS) was used to visually explore the relative (dis)similarity between samples based on the fungal community detected in each. Prior to ordination, samples (or subsets of samples) were normalized to relative read abundance, and ordination based on Bray–Curtis dissimilarity was conducted using the vegan R package (version 2.5–7; [46] with R version 3.6.2 [47] using a maximum of 200 random starts and condensed to two dimensions. Ordination was conducted on beetle samples and phloem samples prior to conducting ordination with samples combined. There were 7155 sequence reads (167 samples, 258 OTUs) and 4807 sequence reads (164 samples, 96 OTUs) included in ordination for the beetle and phloem subsets, respectively. (For an overview of the number of samples representing each sample type, see Table S1).

Permutational multivariate analysis of variance (PERMANOVA) tests were used to test for the significance of observed patterns via the “adonis2” function of the vegan R package, and in some cases homogeneity of dispersion was tested for groups using the “betadisper” function coupled with the base R “anova” function. For both beetle and phloem sample datasets, standard PERMANOVA tests were used to test for a significant difference in groups based on fire history, and both marginal and standard PERMANOVA tests were carried out on the full dataset to investigate the relative significance of differences in fungal community composition based on fire history and beetle vs. phloem samples. We also used PERMANOVA to test for significance of groupings based on sample type (male and female, body and gut) for beetle samples and relating to sampling time and bark beetle colonization status for phloem samples. Pairwise PERMANOVAs with a Bonferroni correction was then used to test for significant differences between uncolonized phloem and first instar colonized phloem, as well as first instar vs. third instar colonized phloem samples. Pairwise PERMANOVA with Bonferroni correction was also used to test for effect of fire history within uncolonized samples, within first instar colonized samples, and within third instar colonized samples.

Based on the results of ordination and PERMANOVA tests, we identified eight groups of samples (four categories of samples from both burnt and unburnt sites): beetle (all beetle samples), uncolonized pine phloem (no larvae; pre-colonization samples, and uncolonized control samples from first and third instar sampling time points combined), pine phloem colonized by first instar larvae, and pine phloem colonized by third instar larvae. For an in-depth distribution analysis of the most prominent OTUs across these eight categories, we identified a core community including OTUs with a minimum of 1% average relative read abundance and detection in a minimum of 30% of the samples in any of the eight groups. A heatmap was used to display the shifts in fungal community across the eight sample groups. Color intensity represents the scaled (but not centered) average relative read abundance within each OTU, and the average relative read abundance is reported within each cell.

Taxonomic assignment of the 15 OTUs identified as the core community was manually curated in October 2021 by running BLAST queries to the online UNITE database [48]. A Unite Species Hypothesis (USH) threshold of 1.5% dissimilarity was selected to reflect the clustering used in this study. Species identification was often not possible since the ITS2 region is highly conserved across multiple closely related species for most of the lineages represented in the core community [49]. The curated taxonomy was used for discussing possible functions and lifestyles represented by the core OTUs in this study system (Table S2).

#### Phloem Chemistry

To analyze phloem chemistry, a partially Bayesian mixed-effects model was fitted for each of the three nutrient ratios (C:N, C:P, N:P) using the blme R package (version 1.0–5; [50]. In each model, fire history (burnt or unburnt site), bark beetle colonization (colonized or uncolonized phloem), and sampling time (first or third instar) were treated as predictor variables and the nutrient ratio was treated as the response variable. Interaction terms for each pair of the three explanatory variables and a three-way interaction were included in the model. The nutrient ratio measured pre-colonization for each tree was treated as a nuisance co-variate, and the interaction between tree and site was treated as a random effects variable. The assumption of equal slopes was tested by comparing the model described here to a model that also includes an interaction between the pre-colonization nutrient ratio and each of fire history, bark beetle colonization, and sampling time. In all cases, the pre-colonization nutrient ratio values were centered (but not scaled), and sum-to-zero contrast coefficients were applied to each of the three explanatory factors. A model including interactions was significantly better for C:N (χ^2^_(3)_ = 9.7412, Pr(> χ^2^) = 0.0209) and for N:P (χ^2^_(3)_ = 17.974, Pr(> χ^2^) = 0.0004) but not for C:P (χ^2^_(3)_ = 6.2103, Pr(> χ^2^) = 0.1018), indicating that the ANCOVA assumption of equal slopes for a co-variate is violated for the C:N and N:P ratio models. The inclusion of interaction terms has little impact in the outcome of the analysis for the C:P ratio model, so the interaction terms were retained for all three models. However, it seems that the interaction between pre-colonization ratio value and sampling time may be driving this, so a model fitted with only the interaction between pre-colonization nutrient ratio and sampling time was fitted to see if the more complex model with all three co-variate–explanatory variable interactions was still significantly better. In all three comparisons, the more complex model was not significantly better (C:N: χ^2^_(2)_ = 0.3075, Pr(> χ^2^ = 0.8575; C:P: χ^2^_(2)_ = 5.8374, Pr(> χ^2^) = 0.054; N:P: χ^2^_(2)_ = 2.4065, Pr(> χ^2^) = 0.3002). Therefore, the final models included the three explanatory variables (bark beetle colonization, fire history, and sampling time) and their interactions, pre-colonization nutrient ratio, the interaction between the pre-colonization nutrient ratio and sampling time, and the interaction between site and tree as a random effects variable.

Assumptions of mixed-effects models were inspected both visually and statistically. We tested for homogeneity of variance by plotting the standardized residuals against predicted values. The data for all three nutrient ratios were mildly heteroscedastic, however reasonably so (data not shown). Levene’s tests indicate that the assumption of equal variance of residuals is violated for several grouping factors across the three models (Tables S3–S5). Normality of errors for both the residuals and random effects were inspected visually by histogram for residuals, Q-Q plots for random effects and statistically via Shapiro–Wilk normality tests. Shapiro–Wilk tests indicate that the distributions of residuals deviate from normality for all three nutrient ratios (Tables S3–S5). Histograms show that the distribution looks close to normal for all three nutrient ratios, with the number of values more than two standard deviations from the median within reason given the number of samples (data not shown). Random effects did not have errors that deviated from a normal distribution for any of the three models, as determined via Q-Q plots (data not shown) and Shapiro–Wilk normality tests (Tables S3–S5). Although these datasets may violate some of these model assumptions, a recent study showed that mixed-effects models are quite robust to such violations [51]. We therefore proceeded with analysis.

Type II Wald χ^2^ tests via the car R package (version 3.0–10; [52] were used to determine the marginal significance of each term (Tables 1, 2, and 3. Significance of each term was also evaluated by 95% confidence intervals (CI; Tables 1, 2, and 3). The performance R package (version 0.7.2; [53] was used to determine the marginal and conditional *R*^2^ calculated using the Nakagawa method, and the marginal and conditional intra-class correlation coefficient (ICC) values for each model. Conditional *R*^2^ values of 0.731, 0.774, and 0.777 for C:N, C:P, and N:P, respectively, show that the model in each case accounts for between 73 and 78% of the variation in the dataset. Marginal *R*^2^ values of 0.469, 0.352, and 0.320 for C:N, C:P, and N:P, respectively, indicate that of the variance in the model, 47%, 35%, and 32% is attributable to the fixed effects. All in all, the model is a good fit for the data in each case, although variation between tree and site (random effects) explains a large proportion of the variance. ICC values confirm this observation of large proportions of variance explainable by random effects for C:N (ICC_adj_ = 0.494, ICC_cond_ = 0.262), C:P (ICC_adj_ = 0.651, ICC_cond_ = 0.422), and N:P (ICC_adj_ = 0.672, ICC_cond_ = 0.457).

**Table 1:** Type II Wald χ^2^ tests and 95% confidence intervals for explanatory variables and interactions in the final C:N ratio Bayesian linear mixed-effects regression model. Significant test results in bold and marked with asterisk, Pr(> χ.^2^) values (≤ 0.05)

Variable	χ^2^_(1)_	Pr(> χ^2^)	2.5%	97.5%
fire history	0.1576	0.6914	− 6.8616	4.4161
**bark beetle colonization**	**51.5438**	< 0.0000*	− 7.7790	− 4.3182
**sampling time**	**160.1106**	< 0.0000*	− 7.9413	− 4.4781
**pre-colonization C:N**	**5.2685**	0.0217*	0.2094	1.3012
**fire history × bark beetle colonization**	**21.3155**	< 0.0000*	2.3967	5.8576
**fire history × sampling time**	**44.9590**	< 0.0000*	− 6.2651	− 2.6732
**bark beetle colonization × sampling time**	**22.4943**	< 0.0000*	− 5.9932	− 2.5300
**sampling time × pre-colonization C:N**	**9.4124**	0.0022*	− 0.3662	− 0.0820
fire history × bark beetle colonization × sampling time	0.7451	0.3880	− 2.5007	0.9625

**Table 2:** Type II Wald χ^2^ tests and 95% confidence intervals for explanatory variables and interactions in the final C:P ratio Bayesian linear mixed-effects regression model. Significant test results in bold and marked with asterisk, Pr(> χ.^2^) values (≤ 0.05)

Variable	χ^2^_(1)_	Pr(> χ^2^)	2.5%	97.5%
fire history	0.3713	0.5423	− 303.3535	145.3486
**bark beetle colonization**	**5.6866**	0.0171*	13.5997	100.4377
**sampling time**	**78.4676**	< 0.0000*	− 113.8983	− 27.0345
pre-colonization C:P	2.5643	0.1093	− 0.0160	0.6810
fire history × bark beetle colonization	0.0402	0.8411	− 46.1861	40.6519
fire history × sampling time	3.2554	0.0712	− 35.4410	64.8213
**bark beetle colonization × sampling time**	**33.0173**	< 0.0000*	− 176.6356	− 89.7751
sampling time × pre-colonization C:P	0.3703	0.5429	− 0.0865	0.0455
**fire history × bark beetle colonization × sampling time**	**14.7234**	0.0001*	− 129.2474	− 42.3869

**Table 3:** Type II Wald χ^2^ tests and 95% confidence intervals for explanatory variables and interactions in the final N:P ratio Bayesian linear mixed-effects regression model. Significant test results in bold and marked with asterisk, Pr(> χ.^2^) values (≤ 0.05)

Variable	χ^2^_(1)_	Pr(> χ^2^)	2.5%	97.5%
fire history	1.6250	0.2024	− 2.5292	0.9616
**bark beetle colonization**	**72.6535**	< 0.0000*	1.1265	1.8083
sampling time	0.2238	0.6361	0.0754	0.7574
pre-colonization N:P	0.8075	0.3689	− 0.1240	0.4150
**fire history × bark beetle colonization**	**23.4658**	< 0.0000*	− 1.1750	− 0.4932
**fire history × sampling time**	**14.2598**	0.0002*	0.7046	1.4620
**bark beetle colonization × sampling time**	**7.5743**	0.0059*	− 0.8671	− 0.1852
**sampling time × pre-colonization N:P**	**15.6855**	0.0001*	0.0506	0.1481
**fire history × bark beetle colonization × sampling time**	**18.7486**	0.0000*	− 1.1007	− 0.4187

The results show that for all three nutrient ratios, bark beetle colonization had a significant main effect, but it also had significant interactions with other explanatory variables, indicating that the significance of bark beetle colonization is dependent on the value of these explanatory variables. Fire history had no significant main effect on any of the three nutrient ratios, but it did have significant interactions with other explanatory variables. This indicates that while fire history alone cannot explain much variation on nutrient ratio, fire history can play a role in determining the effects (either in significance or direction) of other explanatory variables. Each nutrient ratio dataset was therefore subset by fire history, and the same model (excluding the fire history term) was used to tease apart these interactions. For each, type II Wald χ^2^ tests and 95% CIs were used to evaluate the significance of the main and interaction effects for each remaining explanatory variable (sampling time and bark beetle colonization). The six subset models also had similar *R*^2^ and ICC values as the full models (Table S6).

## Results

### Fungal Communities in Bark Beetle and Phloem Sample Subsets

**Fig. 2:**
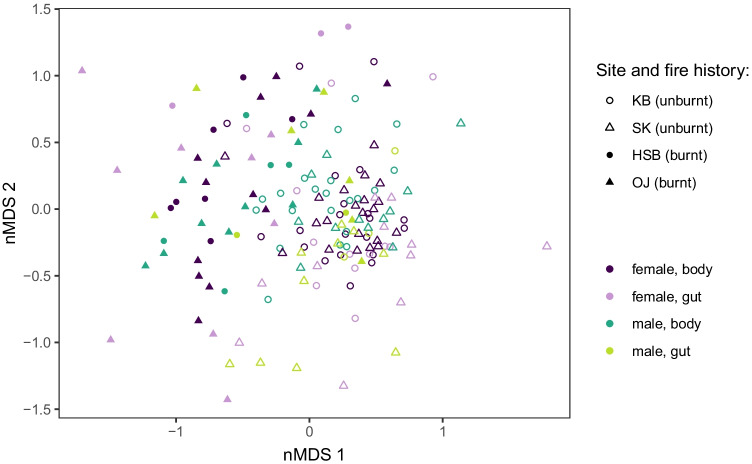
Non-metric multidimensional scaling (nMDS) ordination showing fungal community differentiation in bark beetle samples associated with fire history of sampling locality. Filled symbols represent samples taken from plots that experienced forest fire, and open symbols represent samples from unburnt sites. Plot excludes samples with less than 10 sequence reads. There are 167 samples, 258 OTUs, and 7155 sequence reads represented in the plot. Plot stress = 0.241

**Fig. 3:**
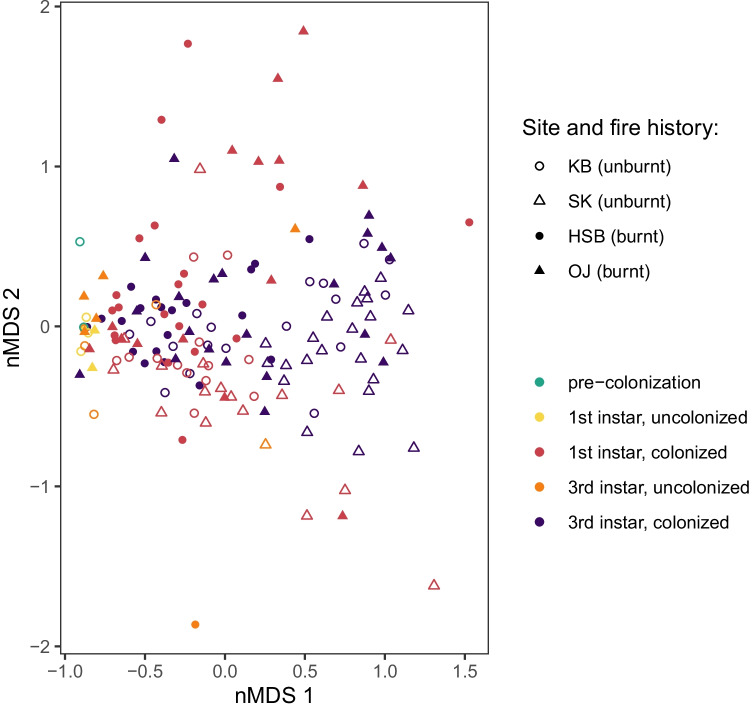
Non-metric multidimensional scaling (nMDS) ordination displaying differentiation of fungal communities in phloem samples based on fire history of sampling locality. Filled symbols represent samples taken from plots that experienced forest fire, and open symbols represent samples from unburnt sites. Plot excludes samples with less than 10 sequence reads. There are 164 samples, 96 OTUs, and 4807 sequences read represented in the plot. Plot stress = 0.186

For fungal communities of both beetle and phloem samples, the most conspicuous pattern observed was a distinction between samples from burnt and unburnt sites (Figs. 2, 3). PERMANOVA tests (permuted within site) indicate that samples from burnt and unburnt sites are significantly different (bark beetle samples: *F*_(1,165)_ = 13.099, *R*^2^ = 0.074, Pr(> *F*) ≤ 0.001; phloem samples: *F*_(1,162)_ = 9.318, *R*^2^ = 0.054, Pr(> *F*) ≤ 0.001). No structuring based on sampling location (i.e., KB vs. SK for unburnt and HSB vs. OJ for burnt; Fig. 2) was observed. There was a significant difference in the dispersion of groups (samples from burnt vs. unburnt sites) for beetle samples (*F* = 22.387, Pr(> *F*) ≤ 0.001) but not for phloem samples (*F* = 0.015, Pr(> *F*) = 0.903). However, it is clear upon visual inspection of beetle sample ordination that the centroids of the groups are different (Fig. 2). Thus, the significant PERMANOVA results are not due to the variation in dispersion.

We did not identify strong patterns in the ordination of beetle samples reflecting sample type (i.e., female vs. male, or body vs. gut) (Fig. 2); however, PERMANOVA indicates that a small amount of variation may be explained by sex (*F*_(1,165)_ = 1.5885, *R*^2^ = 0.0095, Pr(> *F*) = 0.025) and body region (*F*_(1,165)_ = 2.6264, *R*^2^ = 0.0157, Pr(> *F*) = 0.005). The variation explained by sex is negligible (less than 1%), and the relatively small amount variation explainable by body region (1.6%) may be somewhat driven by the marginally significantly different group dispersions (*F* = 3.592, Pr(> *F*) = 0.060). We therefore have omitted these two variables from the remainder of the fungal community analyses and have chosen to group the beetle samples into just two groups based on fire history. No substantial patterns were associated with relationship between body and gut samples that were extracted from the same beetle collection (Fig. S2), so we chose to treat the samples as independent rather than remove data.

For phloem samples, on the other hand, fungal community variation may be related to colonization status and sampling time in addition to fire history (Fig. 3). Ordination revealed a relationship between fungal community composition and the presence or absence of bark beetle colonization as all uncolonized phloem samples, regardless of sampling time, cluster together (Fig. 3). For beetle-colonized phloem samples, fungal communities varied over time from the first instar to the third instar sampling time points (Fig. 3). PERMANOVA supports that samples, when grouped into uncolonized phloem, colonized phloem first instar, and colonized phloem third instar, are significantly different based on this grouping (*F*_(5,158)_ = 7.554, *R*^2^ = 0.1929, Pr(> *F*)_corr_ = 0.005). Pairwise PERMANOVA with Bonferroni correction (permuting within site) supports this pattern, with significant differences between uncolonized samples and first instar colonized samples (*F*_(3,79)_ = 4.577, *R*^2^ = 0.1481, Pr(> *F*)_corr_ = 0.01), and between first and third instar colonized samples (*F*_(3,141)_ = 8.614, *R*^2^ = 0.1549, Pr(> *F*)_corr_ = 0.01). Pairwise PERMANOVA with Bonferroni correction was used to test for variation between samples from burnt vs. unburnt sites within each of these three groups of samples (i.e., uncolonized samples, first instar colonized samples, and third instar colonized samples), and reveals that the uncolonized samples are not significantly different between burnt and unburnt sites (*F*_(1,17)_ = 0.767, *R*^2^ = 0.0432, Pr(> *F*)_corr_ = 1), whereas the difference between burnt and unburnt sites is larger for third instar colonized samples (*F*_(1,79)_ = 10.631, *R*^2^ = 0.1187, Pr(> *F*)_corr_ = 0.003) than for first instar colonized samples (*F*_(1,62)_ = 6.6425, *R*^2^ = 0.0968, Pr(> *F*)_corr_ = 0.003). This pattern is also observable in ordination of phloem samples, where fungal communities found in burnt vs. unburnt sites increasingly diverge over time (Fig. 3).

Based on these results, we grouped samples into eight categories for subsequent analyses of fungal community composition: beetle samples, uncolonized phloem (including controls from both first and third instar sampling times), first instar beetle-infested phloem and third instar beetle-infested phloem, all from burnt and unburnt sampling locations.

### Fungal Communities in All Samples and Core Community

**Fig. 4:**
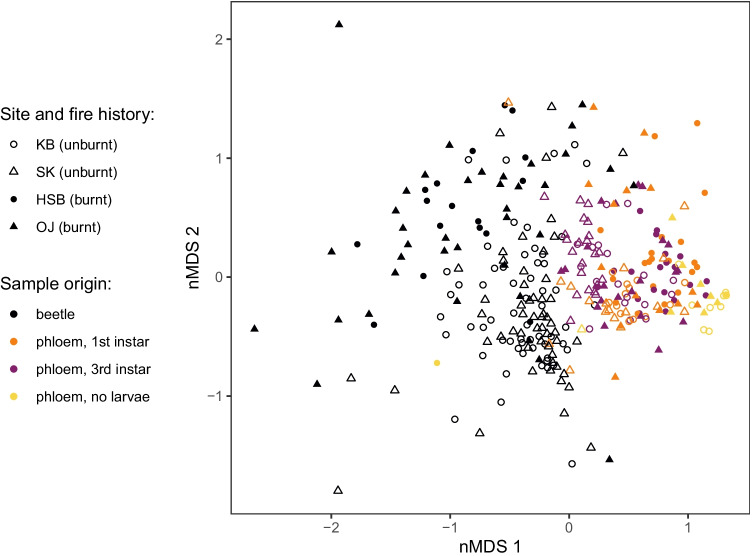
Non-metric multidimensional scaling (nMDS) ordination displaying fungal community shifts associated with forest fire, sampling type, and substrate (phloem vs. beetle samples). Filled symbols represent samples taken from plots that experienced forest fire, and open symbols represent samples from unburnt sites. Plot excludes samples with less than 10 sequence reads. There are 331 samples, 286 OTUs, and 11,962 sequence reads represented in the plot. Plot stress = 0.223

When fungal communities in all samples were visualized together via nMDS ordination, the most striking patterns observed were a distinction between beetle and phloem samples along nMDS 1, and some structuring along nMDS 2 corresponding to fire history (Fig. 4). Fungal communities associated with beetle samples are clearly separated from those in phloem samples (Fig. 4), and this pattern is supported by PERMANOVA (*F*_(1,329)_ = 54.701, *R*^2^ = 0.1426, Pr(> *F*) = 0.005). The clear distinction between samples from burnt vs. unburnt sites is also supported by PERMANOVA (*F*_(1,329)_ = 18.63, *R*^2^ = 0.0536, Pr(> *F*) ≤ 0.001), and a comparison of the marginal variation of the two terms when they are both included in the model indicates that the distinction between phloem and beetle samples is stronger than the difference between samples based on fire history, although both explain a large proportion of variation (fire history: *F*_(1,328)_ = 18.072, *R*^2^ = 0.0448, Pr(> *F*) ≤ 0.001; phloem vs. beetle samples: *F*_(1,328)_ = 53.982, *R*^2^ = 0.1338, Pr(> *F*) ≤ 0.001). Ordination also reveals along nMDS 1 that the phloem samples from the third instar colonized samples are most similar to the beetle samples, whereas the uncolonized phloem samples are the most different from the beetle samples.

**Fig. 5:**
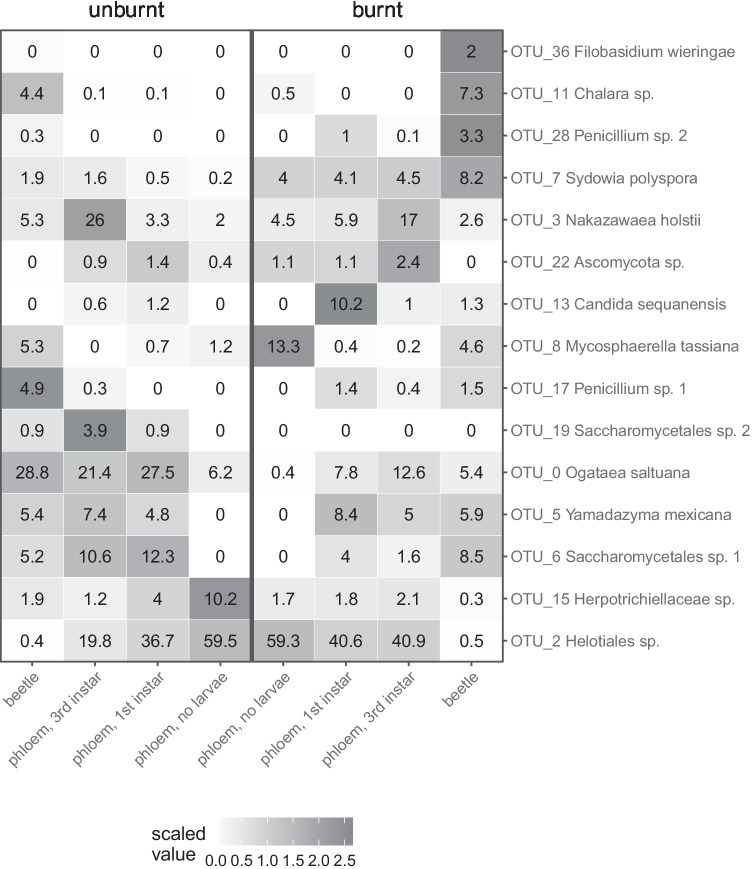
Heatmap of the average relative sequence read abundance across samples for 15 OTUs identified as the core community in this study. To the right, OTU number and assigned taxonomy

The same pattern is observed when analyzing the relative sequence read abundance of the 15 OTUs, identified as the core community which together represent 73% of the reads in the analyzed dataset. A heatmap of the average relative read abundance of each core OTU among the groups of samples reveals an overall difference in fungal community structure between samples from burnt and unburnt sites, except for the uncolonized phloem samples (Fig. 5). Fungal communities from uncolonized phloem samples could not be distinguished based on fire history. Analysis of the core fungal community demonstrates that uncolonized phloem samples from both burnt and unburnt sites were highly dominated (average relative read abundance of 59–60%) by a single taxon, Helotiales sp. (OTU_2) (Fig. 5). In contrast, fungal communities from beetle samples were highly distinct based on fire history (Figs. 2, 5). Strikingly, the yeast *Ogataea saltuana* (OTU_0) made up an average of 29% of the fungal community on/in beetles from unburnt sites compared to 5% on/in beetles from burnt sites (Fig. 5). Similar to the pattern observed in nMDS ordination, beetle-colonized phloem samples (first and third instar) appear as intermediate stages between beetles and uncolonized phloem samples (Fig. 5). While Helotiales sp. (OTU_2) dominated the detected fungal community in uncolonized phloem samples (an average of nearly 60% of the sequence reads), a shift to a community where OTUs associated with beetle samples were represented in higher and higher relative read abundance over time in phloem samples was observed, especially in unburnt sites. Communities of beetle-colonized phloem samples more closely resembled the communities of bark beetle samples, but with communities from burnt and unburnt sites becoming increasingly diverged over time from first to third instar. Another Leotiomycetidae taxon, *Chalara* sp. (OTU_11), made up an average of 4–7% of the fungal community in beetle samples, but less than 1% in phloem samples. Interestingly, *Chalara* sp. was not detected in colonized phloem samples at burnt sites where its relative read abundance on/in beetles was the highest (Fig. 5).

Seven of the 15 core OTUs could be identified to USH with an assigned species name (Table S2), but identification remains uncertain since species boundaries may be blurred by clustering and short sequence length with low resolution for the detected fungal lineages. Six of the core OTUs represent yeasts in the order Saccharomycetales (OTU_0, OTU_3, OTU_5, OTU_6, OTU_13, and OTU_19). Of the Saccharomycetales OTUs, only *O. saltuana* (OTU_0) and *Nakazawaea holstii* (OTU_3) were detected in the uncolonized phloem of both burnt and unburnt sites. Both increased in average relative read abundance over time in beetle-colonized phloem (Fig. 5). The remaining Saccharomycetales OTUs were not detected in uncolonized phloem and only found in beetle-associated samples. Saccharomycetales sp. 2 (OTU_19) was only found in beetle samples and beetle-colonized phloem from unburnt sites (Fig. 5). The basidiomycetous yeast *Filobasidium wieringae* was detected only in beetle samples from burnt sites.

Two OTUs identified to genus *Penicillium* (OTU_17 and OTU_28) comprised up to 5% of the fungal community associated with beetle samples. While neither were detected in uncolonized phloem samples, both were found at low relative read abundance in beetle-colonized phloem samples from burnt sites (Fig. 5). Two OTUs (OTU_7 and OTU_8) in the sub-class Dothideomycetidae were detected in all (OTU7) or neary all (OTU8) sample types. One of them, OTU_7, was identified as the pine pathogenic fungi *Sydowia polyspora* and made up a larger proportion of the fungal community (4–8%) of beetle-colonized phloem and beetle samples from burnt sites compared to samples from unburnt sites, where this OTU made up less than 2% (Fig. 5).

### Phloem Chemistry

**Fig. 6:**
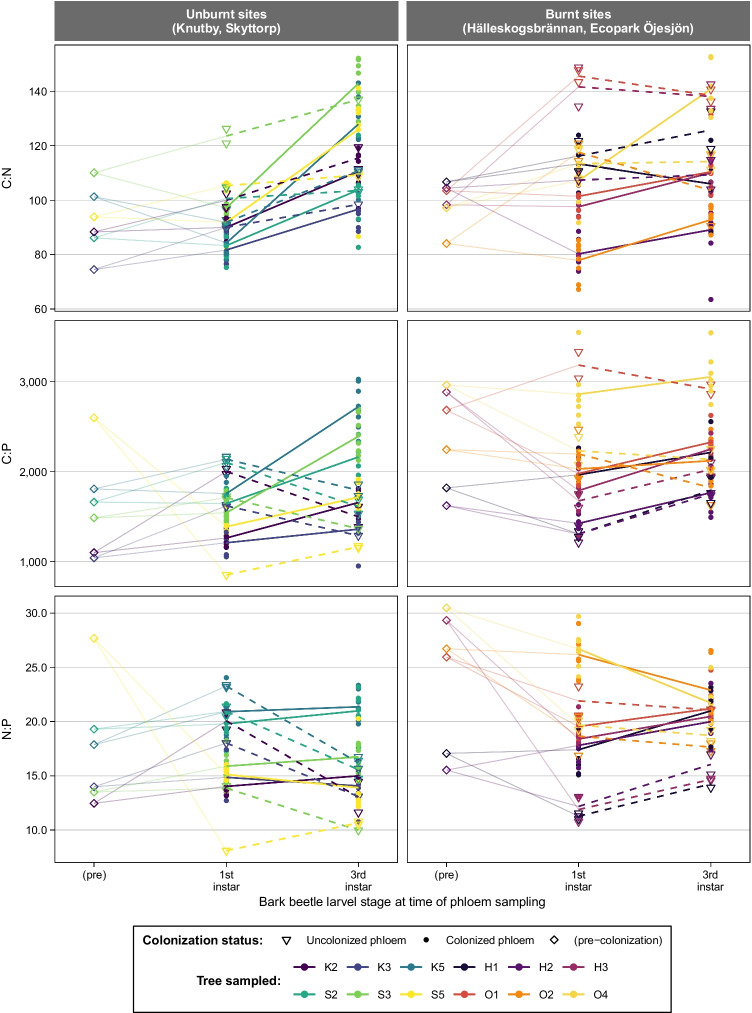
C:N, C:P, and N:P ratios (molar) for all samples. Lines indicate mean ratio value for colonized (solid line) and uncolonized (dashed line) phloem samples for each tree. Faint line connects the pre-colonization value to both the colonized (closed symbols) and uncolonized (open symbols) value for each tree. See Fig. S2 to examine values for samples from each tree separately, and Fig. S3 for model-predicted values plotted

Across all sites, there was a general trend of decreasing nutrient quality, i.e., increasing C:N and C:P ratios, over time from pre-colonization to third instar gallery samples (Fig. 6). The five terms (bark beetle colonization, sampling time, the interaction between the two, the nutrient ratio measured pre-colonization, and the interaction between the pre-colonization ratio and sampling time) included in each of the six subset models are significant in different combinations for each model (Tables 4, 5, 6, 7, 8, and 9). For C:N ratio, sampling time, bark beetle colonization, and their interaction were all significant in both burnt and unburnt subset models, and the pre-colonization C:N main effect and its interaction with sampling time were also significant in the unburnt subset (Tables 4 and 5). In the C:N burnt model, bark beetle colonization had the largest effect, and sampling time had the largest effect in the C:N unburnt subset model (Tables 4 and 5). For the C:P ratio models, sampling time was the only variable with a significant effect in the burnt model, whereas sampling time and the interaction between sampling time and bark beetle colonization were also significant in the unburnt C:P model (Tables 6 and 7). And finally, for the N:P models, a main effect of bark beetle colonization was significant in both burnt and unburnt models, but the combination of the rest of the terms varies between the two (Tables 8 and 9). Bark beetle colonization has the largest effect in the burnt N:P model, whereas bark beetle colonization–sampling time interaction had the most significant effect in the unburnt N:P model (Tables 8 and 9). Taken together, this indicates that the three nutrient ratios, C:N, C:P, and N:P, are affected differently by bark beetle colonization, sampling time, and pre-colonization value, and this system is different for sites that have had a recent forest fire than for sites that have not. Fire history, beetle colonization, larval stage of the bark beetles, and initial nutrient ratios prior to bark beetle infestation all play roles in driving phloem nutrient in general. However, in a complex relationship where the effects of factors may depend on values of other variables, they may vary in intensity and direction with respect to these other factors. Some of these relationships are more easily recognized when the data is visualized, either using the actual response values (Fig. 6, Fig. S3), or by inspecting a plot of the predicted values (Fig. S4).

**Table 4:** Type II Wald χ^2^ tests and 95% confidence intervals for explanatory variables and interactions in the final C:N ratio for samples from burnt sites Bayesian linear mixed-effects regression model. Significant test results in bold and marked with asterisk, Pr(> χ.^2^) values (≤ 0.05)

Variable	χ^2^_(1)_	Pr(> χ^2^)	2.5%	97.5%
**bark beetle colonization**	**59.7424**	< 0.0000*	− 12.7496	− 7.6019
**sampling time**	**18.8727**	0.0000*	− 5.0538	0.0940
pre-colonization C:N	0.3335	0.5636	− 0.8805	1.9979
**bark beetle colonization × sampling time**	**7.0376**	0.0080*	− 6.0664	− 0.9186
sampling time × pre-colonization C:N	2.7833	0.0953	− 0.0403	0.5091

**Table 5:** Type II Wald χ^2^ tests and 95% confidence intervals for explanatory variables and interactions in the final C:N ratio for samples from unburnt sites Bayesian linear mixed-effects regression model. Significant test results in bold and marked with asterisk, Pr(> χ.^2^) values (≤ 0.05)

Variable	χ^2^_(1)_	Pr(> χ^2^)	2.5%	97.5%
bark beetle colonization	**4.003**	0.0454*	− 3.9318	0.1944
sampling time	**265.206**	< 0.0000*	− 11.9815	− 7.8535
pre-colonization C:N	**16.026**	0.0000*	0.5514	1.1491
bark beetle colonization × sampling time	**22.664**	< 0.0000*	− 7.0953	− 2.9674
sampling time × pre-colonization C:P	**36.421**	< 0.0000*	− 0.5743	− 0.2929

**Table 6:** Type II Wald χ^2^ tests and 95% confidence intervals for explanatory variables and interactions in the final C:P ratio for samples from burnt sites Bayesian linear mixed-effects regression model. Significant test results in bold and marked with asterisk, Pr(> χ.^2^) values (≤ 0.05)

Variable	χ^2^_(1)_	Pr(> χ^2^)	2.5%	97.5%
bark beetle colonization	3.0299	0.0817	− 7.3695	126.9412
**sampling time**	**19.4656**	0.0000*	− 159.9512	− 25.6405
pre-colonization C:P	3.5148	0.0608	0.1167	0.9988
bark beetle colonization × sampling time	1.9036	0.1677	− 114.5436	19.7671
sampling time × pre-colonization C:P	0.1096	0.7406	− 0.0866	0.1220

**Table 7:** Type II Wald χ^2^ tests and 95% confidence intervals for explanatory variables and interactions in the final C:P ratio for samples from unburnt sites Bayesian linear mixed-effects regression model. Significant test results in bold and marked with asterisk, Pr(> χ.^2^) values (≤ 0.05)

Variable	χ^2^_(1)_	Pr(> χ^2^)	2.5%	97.5%
bark beetle colonization	2.9804	0.0843	2.1180	110.0165
**sampling time**	**80.2984**	< 0.0000*	− 102.4289	5.4198
pre-colonization C:P	0.0930	0.7605	− 0.4326	0.6517
**bark beetle colonization × sampling time**	**62.6113**	< 0.0000*	− 272.2899	− 164.4388
sampling time × pre-colonization C:P	2.1756	0.1402	− 0.1406	0.0200

**Table 8:** Type II Wald χ^2^ tests and 95% confidence intervals for explanatory variables and interactions in the final N:P ratio for samples from burnt sites Bayesian linear mixed-effects regression model. Significant test results in bold and marked with asterisk, Pr(> χ.^2^) values (≤ 0.05)

Variable	χ^2^_(1)_	Pr(> χ^2^)	2.5%	97.5%
**bark beetle colonization**	**78.4014**	< 0.0000*	1.7933	2.8097
sampling time	0.8924	0.3448	− 0.8448	0.1717
pre-colonization N:P	1.7991	0.1798	− 0.0276	0.5535
bark beetle colonization × sampling time	0.8073	0.3689	− 0.2747	0.7417
**sampling time × pre-colonization N:P**	**21.2492**	< 0.0000*	0.0953	0.2356

**Table 9:** Type II Wald χ^2^ tests and 95% confidence intervals for explanatory variables and interactions in the final N:P ratio for samples from unburnt sites Bayesian linear mixed-effects regression model. Significant test results in bold and marked with asterisk, Pr(> χ.^2^) values (≤ 0.05)

Variable	χ^2^_(1)_	Pr(> χ^2^)	2.5%	97.5%
**bark beetle colonization**	**8.1073**	0.0044*	0.2443	1.1024
**sampling time**	**3.9313**	0.0474*	0.7273	1.5849
pre-colonization N:P	0.0002	0.9885	− 0.5051	0.4960
**bark beetle colonization × sampling time**	**34.0067**	< 0.0000*	− 1.7078	− 0.8502
sampling time × pre-colonization N:P	0.1284	0.7201	− 0.0522	0.0761

## Discussion

In our study system, we find that forest fire significantly impacts the fungal community composition associated with *T. piniperda*, and that fire may also indirectly impact nutrient availability in fungal-colonized phloem of beetle galleries. However, the substrate quality of the phloem in beetle galleries in general decreased over time as indicated by increasing C:N and C:P ratios (Fig. 6, Figs. S3 and S4). This is in contrast to observations of the mycangial bark beetle species *Dendroctonus brevicomis* where the specifically associated fungi have been found to transport nutrient from the inner wood out to the phloem, thereby improving substrate quality for the growing larvae [11, 15]. *T. piniperda*, on the other hand, has no specific body structure to carry fungal propagules, such as mycangia or hairs, for active vectoring of fungi to newly colonized trees. In accordance with this, we find no pattern of specific fungal community associated with the outside of beetles compared to the fungal community recovered from beetle gut samples (Fig. 2). Minimal differences were detected in the fungal communities associated with beetle males compared to females, further indicating that vectoring of specific fungal communities is not a strategy associated with the *T. piniperda* life cycle. Together with decreasing substrate quality in galleries, these results strongly indicate that. *T. piniperda* does not benefit directly from fungal communities. However, it is important to remember that the low sequencing depth of several samples in the current study may have hampered our ability to detect specific fungal–beetle associations, in particular for rare taxa (in terms of low relative abundance).

Despite the lack of specificity, we show that fungi are transmitted by the beetle as indicated by our observation that beetle-associated fungal communities are more similar to those in galleries at the third instar compared to those in uncolonized phloem samples (Fig. 4). The trend is more pronounced in unburnt sites compared to burnt sites, suggesting that phloem fungal communities in unburnt sites are derived to a larger extent from the community introduced by beetles upon colonization. Several of the yeast fungi belonging to the class Saccharomycetes have previously been shown to be necessary for some insects to degrade wood sugars as a source of nutrients, for detoxifying tree defense, and for protection from biotic stresses [21, 54–56]. In our study, OTUs assigned to Saccharomycetes made up a considerable fraction of the fungal community in phloem samples suggesting that they may play similar roles in this system. In burnt sites, on the other hand, members of the pre-colonization community, i.e., Helotiales sp. (OTU_2), remain dominant in third instar galleries. The abundant taxon Helotiales sp. (OTU_2) was detected at low abundance in beetles while dominating the community of uncolonized phloem, at both burnt and unburnt sites. Together, our results suggest that the event of forest fire affects the developmental trajectory of fungal community composition following bark beetle colonization.

Interestingly, only few of the taxa identified in the core fungal community represent organisms that have previously been observed in association with *T. piniperda*. For example, the pine pathogenic fungus *S. polyspora* was detected in our core community and is one of the fungal species previously reported in association with *T. piniperda* in Sweden [37, 57, 58]. Curiously, other species found in association with *T. piniperda* in earlier studies, *Leptographium wingfieldii* and *Ophiostoma minus*, were not detected in our study. Their absence in the samples indicates that these species are either absent from the sites or present at too low abundance to be detected with the current sampling and sequencing depth. *S. polyspora* was also detected in uncolonized wood, and it appears that these fungi are well adapted for growth in the nutrient poor phloem and have strategies that allow them to be vectored by a range of beetles to colonize new substrates. For instance, the potential of *T. piniperda* to vector particularly damaging fungi like *S. polyspora* or invasive fungal species like *Fusarium circinatum* has been shown in other systems [59]. Among published sequences that cluster in the USH identified to genus *Penicillium*, corresponding to OTU_17 and OTU_28, there are several sequences originating from beetles, further indicating that our study captures a typical fungal community that may benefit from the unspecific association with beetles that can vector them to new substrates.

## Supplementary Information

Below is the link to the electronic supplementary material.

Supplementary file1 (DOCX 272 kb)

Supplementary file2 (DOCX 33 kb)

## Data Availability

FASTQ sequences, tagged primer sequences, and the output files from the SCATA pipeline are archived and made available through a FigShare repository at 10.17044/scilifelab.20289000. Furthermore, sample metadata, OTU representative sequences, SINTAX taxonomy prediction results, and the sample by OTU matrix files are also found in the same repository, together with a R Markdown file and associated R scripts to facilitate the reproducibility of all analyses conducted as a part of this study.
