# Supplementary material for: Forest Fire Influence on Tomicus piniperda-Associated Fungal Communities and Phloem Nutrient Availability of Colonized Pinus sylvestris
Source: Microb Ecol. 2022 Jul 13;86(1):224–39. doi: 10.1007/s00248-022-02066-w (PMC10293462; doi:10.1007/s00248-022-02066-w)
Supplement: Supplementary file 1 — Supplementary file1 (DOCX 272 kb) [file 248_2022_2066_MOESM1_ESM.docx]

**Supplementary figures**

Title: Forest fire influence on Tomicus piniperda-associated fungal communities and phloem nutrient availability of colonized Pinus sylvestris

Authors: Kluting, Kerri^1^*, Strid, Ylva^1^*, Six, Diana^2^, Rosling, Anna^1^**

**Figure S1.** Artificial cone used to provide shelter for beetles in collection cups.

**Figure S2.** Plot from Fig. 2 with lines connecting body and gut samples from each individual.

**Figure S3.** C:N, C:P, and N:P ratios for each tree separately

**Figure S4.** Predicted C:N, C:P and N:P ratios, partially Bayesian linear mixed-effects models.


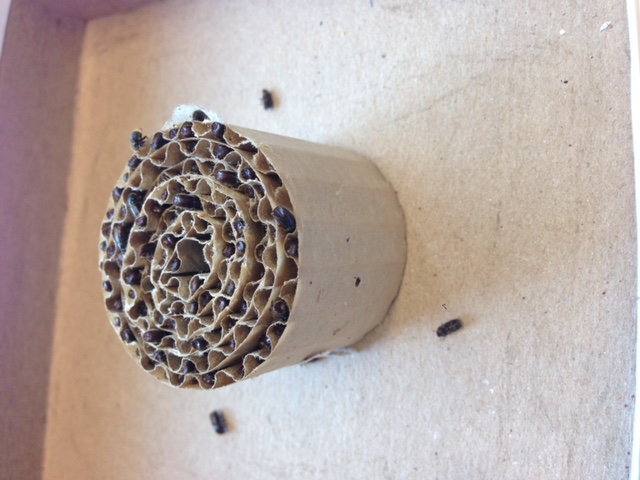


**Figure S1.** Artificial cone used to provide shelter for beetles in collection cups.

**Figure S2.** Plot from Fig. 2 with lines added to connect body and gut samples that originate from the same individual beetle sample, demonstrating that there is not a pattern driven by the non-independence of the samples.

**Figure S3.** C:N, C:P, and N:P ratios for each tree separately. See Fig. 6.

**Figure S4.** Predicted C:N, C:P and N:P ratios based on partially Bayesian linear mixed-effects models. Measured C:N, C:P and N:P ratio values for each sample are plotted in Fig. 6 and Fig. S2.
