# Supplementary material for: Forest Fire Influence on Tomicus piniperda-Associated Fungal Communities and Phloem Nutrient Availability of Colonized Pinus sylvestris
Source: Microb Ecol. 2022 Jul 13;86(1):224–39. doi: 10.1007/s00248-022-02066-w (PMC10293462; doi:10.1007/s00248-022-02066-w)
Supplement: Supplementary file 2 — Supplementary file2 (DOCX 33 kb) [file 248_2022_2066_MOESM2_ESM.docx]

**Supplementary tables**

Title: Forest fire influence on Tomicus piniperda-associated fungal communities and phloem nutrient availability of colonized Pinus sylvestris

Authors: Kluting, Kerri^1^*, Strid, Ylva^1^*, Six, Diana^2^, Rosling, Anna^1^**

**Table S1.** Number of samples in nMDS and heatmap of core OTUs for each sample type and locality

**Table S2.** Taxonomic assignment for 15 core OTUs.

**Table S3.** Test for homogeneity of variance and normality of errors for final C:N ratio.

**Table S4.** Test for homogeneity of variance and normality of errors for final C:P ratio.

**Table S5.** Test for homogeneity of variance and normality of errors for final N:P ratio.

**Table S6.** Bayesian linear mixed-effects regression models for subsets based on nutrient ratio and fire history.

| **Table S1.** Number of samples included in nMDS ordination and heatmap of core OTUs from each sample type and each collection locality. [region = DNA extracted from either body wash or gut; sampling time = larval instar stage of *Tomicus piniperda* at time of sample collection; HSB = Hälleskogsbrännan; OJ = Ecopark Öjesjön; KB = Knutby; SK = Skyttorp] | | | | | | | | | | | | | | | | | |
| --- | --- | --- | --- | --- | --- | --- | --- | --- | --- | --- | --- | --- | --- | --- | --- | --- | --- |
| **Sample type** | |  | **Collection locality and fire history** | | | | | | | | | | | | | | |
|  |  |  |  |  |  |  |  |  |  |  |  |  |  |  |  |  |  |
| **Bark beetle** | |  | **Burnt sites** | | | | | | |  | **Unburnt sites** | | | | | | |
| **beetle sex** | **region** |  | **HSB** | | |  | **OJ** | | |  | **KB** | | |  | **SK** | | |
| female | body |  | 7 | | |  | 12 | | |  | 21 | | |  | 21 | | |
| female | gut |  | 3 | | |  | 9 | | |  | 12 | | |  | 11 | | |
| male | body |  | 5 | | |  | 10 | | |  | 22 | | |  | 12 | | |
| male | gut |  | 2 | | |  | 7 | | |  | 3 | | |  | 10 | | |
|  |  |  |  |  |  |  |  |  |  |  |  |  |  |  |  |  |  |
| **Phloem tissue** | |  | **Trees from burnt sites** | | | | | | |  | **Trees from unburnt sites** | | | | | | |
| **sampling time (larval instar)** | ***T. piniperda* colonization** |  | **H1** | **H2** | **H3** |  | **O1** | **O2** | **O4** |  | **K2** | **K3** | **K5** |  | **S2** | **S3** | **S5** |
| pre-colonization | - |  | 0 | 0 | 0 |  | 0 | 0 | 0 |  | 0 | 1 | 1 |  | 0 | 0 | 0 |
| first | - |  | 0 | 0 | 1 |  | 1 | 0 | 1 |  | 1 | 1 | 1 |  | 0 | 0 | 0 |
| first | + |  | 8 | 7 | 5 |  | 1 | 5 | 8 |  | 5 | 6 | 2 |  | 4 | 6 | 7 |
| third | - |  | 1 | 0 | 0 |  | 2 | 1 | 2 |  | 2 | 2 | 0 |  | 0 | 1 | 0 |
| third | + |  | 6 | 8 | 6 |  | 7 | 6 | 8 |  | 5 | 6 | 7 |  | 8 | 7 | 7 |

| **Table S2.** Taxonomic assignment for 15 core OTUs. BLAST queries to the online UNITE database were conducted 2 Oct 2021. | | | | | | |  |
| --- | --- | --- | --- | --- | --- | --- | --- |
| **Class/OTU** | **Assigned taxon name** | **Best match in UNITE** | **% ID** | **Accession No.** | **UNITE SH** | **Threshold (%)** | **Other sequenes in UNITE SH include:** |
| **Ascomycota** |  |  |  |  |  |  |  |
| Saccharomycetes |  |  |  |  |  |  |  |
| OTU 0 | *Ogataea saltuana* | *Ogataea saltuana* | 100 | JF430478.1 | SH1578026.08FU | 1,5 | yeast, includes association with pine |
| OTU 3 | *Nakazawaea holstii* | *Nakazawaea holstii* | 100 | KY104368.1 | SH1578346.08FU | 1,5 | yeast, includes association with beetles and gallaries |
| OTU 5 | *Yamadazyma mexicana* | *Yamadazyma mexicana* | 99,69 | MG871187.1 | SH1539875.08FU | 1,5 | yeast, includes association with beetles and gallaries |
| OTU 6 | Saccharomycetales sp. 1 | *Candida fructus* | 100 | KT958534 | SH2699462.08FU | 1,5 | yeast and symbionts of parasitic bark beetles |
| OTU 13 | *Candida sequanensis* | *Candida sequanensis* | 100 | FM178365.1 | SH2699217.08FU | 1,5 | yeast from human and *Ciona intestinalis* |
| OTU 19 | Saccharomycetales sp. 2 | Saccharomycetales | 100 | KJ512850 | SH1578353.08FU | 1,5 | pine association, possible *Nakazawaea* |
| Eurotiomycetes |  |  |  |  |  |  |  |
| OTU 15 | Herpotrichiellaceae sp. | Env. Herpotrichiellaceae | 100 | UDB0667708 | SH1565914.08FU | 1,5 | wood-inhabiting and possibly associated with tree pathgens |
| OTU 17 | *Penicillium* sp. 1 | *Penicillium brevicompactum* | 100 | MN105533.1 | SH1529989.08FU | 1,5 | widespread fungi, includes plant associations |
| OTU 28 | *Penicillium* sp. 2 | *Penicillium glabrum* | 100 | MT028087 | SH1529988.08FU | 1,5 | widespread fungi, includes plant associations |
| Dothideomycetes |  |  |  |  |  |  |  |
| OTU 7 | *Hormonema macrosporum* (*S. polyspora*) | *Sydowia polyspora* | 100 | MN200212.1 | SH1515061.08FU | 1,5 | wood-associated fungi, often pine or spuce |
| OTU 8 | *Mycosphaerella tassiana* | Env. *Cladosporium* sp. | 100 | UDB086533 | SH1572792.08FU | 1,5 | widespread fungi, includes plant associations |
| Leotiomycetes |  |  |  |  |  |  |  |
| OTU 2 | Helotiales sp. | Env. Helotiales | 92,96 | UDB079248 | SH2724315.08FU | 1,5 | wood- and leaf-associated yeast |
| OTU 11 | *Chalara* sp. | *Chalara* sp. | 100 | AY590788.1 | SH1522515.08FU | 1,5 | wood-inhabiting and possibly associated with tree pathgens |
| unknown |  |  |  |  |  |  |  |
| OTU 22 | Ascomycota sp. | Fungi | 86,79 | KX195607.1 | — | — |  |
| **Basidiomycota** |  |  |  |  |  |  |  |
| Tremellomycetes |  |  |  |  |  |  |  |
| OTU 36 | *Filobasidium wieringae* | *Filobasidium wieringae* | 100 | MW019466 | SH1631613.08FU | 1,5 | widespread yeast associated with litter and decay |

| **Table S3.** Test for homogeneity of variance for grouping factors and tests for normality of errors for the final C:N ratio Bayesian linear mixed-effects regression model. Significant test results in bold and marked with asterisk, *p* or Pr(>*F*) values (≤ 0.05). | | | | |
| --- | --- | --- | --- | --- |
| **Assumption tested** | **Variable** | **Test** | **Statistic** | **Significance** |
| **homogeneity of variance** | **site** | **Levene's** | ***F*_(3,233)_ = 2.7622** | **Pr(>*F*) = 0.0428*** |
| **homogeneity of variance** | **tree** | **Levene's** | ***F*_(11,225)_ = 3.9057** | **Pr(>*F*) = 0.0000*** |
| homogeneity of variance | bark beetle colonization | Levene's | *F*_(1,235)_ = 0.7226 | Pr(>*F*) = 0.3962 |
| **homogeneity of variance** | **sampling time (1st and 3rd instar)** | **Levene's** | ***F*_(1,235)_ = 6.7237** | **Pr(>*F*) = 0.0101*** |
| **normality of errors** | **residuals** | **Shapiro-Wilk** | ***W* = 0.98414** | ***p* = 0.0096***** |
| normality of errors | random effects | Shapiro-Wilk | *W* = 0.90692 | *p* = 0.1948 |

| **Table S4.** Test for homogeneity of variance for grouping factors and tests for normality of errors for the final C:P ratio Bayesian linear mixed-effects regression model. Significant test results in bold and marked with asterisk, *p* or Pr(>*F*) values (≤ 0.05). | | | | |
| --- | --- | --- | --- | --- |
| **Assumption tested** | **Variable** | **Test** | **Statistic** | **Significance** |
| **homogeneity of variance** | **site** | **Levene's** | ***F*_(3,233)_ = 4.1986** | **Pr(>*F*) = 0.0064*** |
| homogeneity of variance | tree | Levene's | *F*_(11,225)_ = 1.1438 | Pr(>*F*) = 0.3283 |
| **homogeneity of variance** | **bark beetle colonization** | **Levene's** | ***F*_(1,235)_ = 20.811** | **Pr(>*F*) < 0.0000*** |
| homogeneity of variance | sampling time (1st and 3rd instar) | Levene's | *F*_(1,235)_ = 0 | Pr(>*F*) = 0.998 |
| **normality of errors** | **residuals** | **Shapiro-Wilk** | ***W* = 0.96513** | ***p* = 0.0000*** |
| normality of errors | random effects | Shapiro-Wilk | *W* = 0.97804 | *p* = 0.9746 |
| **Table S5.** Test for homogeneity of variance for grouping factors and tests for normality of errors for the final N:P ratio Bayesian linear mixed-effects regression model. Significant test results in bold and marked with asterisk, *p* or Pr(>*F*) values (≤ 0.05). | | | | |
| **Assumption tested** | **Variable** | **Test** | **Statistic** | **Significance** |
| **homogeneity of variance** | **site** | **Levene's** | ***F*_(3,233)_ = 4.6263** | **Pr(>*F*) = 0.0037*** |
| homogeneity of variance | tree | Levene's | *F*_(11,225)_ = 1.4346 | Pr(>*F*) = 0.1585 |
| **homogeneity of variance** | **bark beetle colonization** | **Levene's** | ***F*_(1,235)_ = 4.0556** | **Pr(>*F*) = 0.0452*** |
| homogeneity of variance | sampling time (1st and 3rd instar) | Levene's | *F*_(1,235)_ = 0.132 | Pr(>*F*) = 0.7167 |
| **normality of errors** | **residuals** | **Shapiro-Wilk** | ***W* = 0.98206** | ***p* = 0.0043*** |
| normality of errors | random effects | Shapiro-Wilk | *W* = 0.95388 | *p* = 0.6942 |

| **Table S6.** Conditional *R*^2^, marginal *R*^2^, adjusted Intraclass Correlation Coefficient (ICC), and conditional ICC for the Bayesian linear mixed-effects regression models for subsets based on nutrient ratio and fire history. | | | | |
| --- | --- | --- | --- | --- |
| **Data subset** | ***R*^2^_conditional_** | ***R*^2^_marginal_** | **ICC_adjusted_** | **ICC_conditional_** |
| C:N, burnt sites | 0,762 | 0,209 | 0,699 | 0,553 |
| C:N, unburnt sites | 0,814 | 0,742 | 0,277 | 0,072 |
| C:P, burnt sites | 0,724 | 0,310 | 0,601 | 0,415 |
| C:P, unburnt sites | 0,843 | 0,209 | 0,802 | 0,634 |
| N:P, burnt sites | 0,733 | 0,348 | 0,591 | 0,385 |
| N:P, unburnt sites | 0,854 | 0,059 | 0,844 | 0,795 |
